# Associations Between Sub-Clinical Markers of Cardiometabolic Risk and Exposure to Residential Indoor Air Pollutants in Healthy Adults in Perth, Western Australia: A Study Protocol

**DOI:** 10.3390/ijerph16193548

**Published:** 2019-09-22

**Authors:** Suzanne E. Gilbey, Christopher M. Reid, Rachel R. Huxley, Mario J. Soares, Yun Zhao, Krassi Rumchev

**Affiliations:** 1 School of Public Health, Curtin University, Perth, WA 6148, Australia; sue.gilbey@curtin.edu.au (S.E.G.); Christopher.Reid@curtin.edu.au (C.M.R.); R.Huxley@latrobe.edu.au (R.R.H.); m.soares@curtin.edu.au (M.J.S.); Y.Zhao@curtin.edu.au (Y.Z.); 2 School of Public Health and Preventative Medicine, Monash University, Melbourne, VIC 3800, Australia; 3 College of Science, La Trobe University, Melbourne, VIC 3086, Australia

**Keywords:** Indoor air pollution, cardiovascular, metabolic, blood pressure, risk factors, diabetes mellitus

## Abstract

Background: A growing body of epidemiological and clinical evidence has implicated air pollution as an emerging risk factor for cardiometabolic disease. Whilst individuals spend up to two-thirds of daily time in their domestic residential environment, very few studies have been designed to objectively measure the sub-clinical markers of cardiometabolic risk with exposure to domestic indoor air pollutants. This cross-sectional study aims to investigate associations between the components of domestic indoor air quality and selected sub-clinical cardiometabolic risk factors in a cohort of healthy adults living in Perth, Western Australia. Methods: One hundred and eleven non-smoking adults (65% female) living in non-smoking households who were aged between 35–69 years were recruited for the project. Study subjects were invited to participate in all sections of the study, which included: Domestic indoor air monitoring along with the concurrent 24 h ambulatory monitoring of peripheral and central blood pressure and measures of central hemodynamic indices, standardized questionnaires on aspects relating to current health status and the domestic environment, a 24 h time-activity diary during the monitoring period, and clinic-based health assessment involving collection of blood and urine biomarkers for lipid and glucose profiles, as well as measures of renal function and an analysis of central pulse wave and pulse wave velocity. Results: This study provides a standardized approach to the study of sub-clinical cardiometabolic health effects that are related to the exposure to indoor air pollution. Conclusion: The findings of this study may provide direction for future research that will further contribute to our understanding of the relationship that exists between indoor air pollution and sub-clinical markers of cardiometabolic risk.

## 1. Introduction

In recent years, exposure to environmental pollutants has been hypothesized as having an important role in the development and severity of cardiometabolic disease (CMD) [1,2,3], with studies suggesting that exposure to ambient air pollution might adversely affect a range of sub-clinical cardiometabolic endpoints including blood pressure (BP), glucose and lipid metabolism, and vascular function [4,5,6,7,8,9,10].

Ambient air pollution is composed of complex mixtures of contaminants including particulate matter (PM), gases (e.g., nitrogen oxides, carbon monoxide, and sulphur dioxide) and volatile organic compounds (e.g., formaldehyde) [11,12]. PM itself is a combination of a multitude of solid particles from many sources that differ in size and chemical composition. It is also the portion of air pollution most consistently held responsible for the majority of pollution-related health effects [13]. PM is commonly classified by its aerodynamic diameter: ≤ 10 μm (“thoracic” particles or PM_10_), 10–2.5 μm (“coarse” particles or PM_10–2.5_), ≤2.5 μm (“fine” particles or PM_2.5_) and <0.1 μm (“ultrafine” particles—UFP). Health problems related to exposure to PM have been reported to be directly linked to their size [14].

Previous studies have reported BP increases high enough to trigger cardiovascular events such as strokes and heart failure following ambient air pollution exposures [15,16,17,18,19]; however, findings have been inconsistent [20,21]. Though there have been few, other studies have investigated hemodynamic parameters related to BP that quantify cardiovascular function [19]. Of these limited studies, significant positive associations have been reported between ambient [6,7] and household air pollution [22] exposure, with impaired hemodynamic measures including mean arterial pressure (a surrogate measure for tissue and organ perfusion pressure), augmentation index (a correlate of arterial stiffness and wave reflection), pulse pressure and pulse wave velocity (measures of arterial stiffness). 

Additionally, several recent studies in developing countries have evaluated the relationship between ambient and household air pollution with dyslipidemia (abnormal blood lipid levels) [8,9,23,24,25,26,27]. Dyslipidemia is closely linked to the development and progression of atherosclerosis, and positive associations have been reported between elevated levels of air pollution with total cholesterol [8,9,24], triglycerides [8,9,23,26], and low density lipoproteins [8,9,26]. However, findings have been inconsistent across all studies [25,27]. 

Similar mixed results have been observed where the effects of air pollution exposure have been examined with glucose homeostatic measures such as fasting glucose and glycosated hemoglobin (Hb1Ac) levels [23,24,26,28,29]. HbA1c is a well acknowledged biomarker that reflects average plasma glucose levels over the previous six-to-eight weeks, and an elevated HbA1c level denotes an increased risk of developing diabetes [28]. In a cross-sectional study by Liu et al. [28], positive associations were reported between higher levels of ambient air pollution and both fasting glucose and HbA1c. In contrast, Chuang and colleagues [23] reported an association between higher levels of ambient PM air pollution and HbA1c but not with fasting glucose. Additionally, in a study of Honduran women, positive associations were observed between HbA1c and elevated concentrations of household air pollution from biomass cookstoves [29].

Whilst most published studies of human exposure have related to ambient air pollution, it is well recognized that exposure to air pollution occurs in both outdoor and indoor environments [12], with indoor air quality issues becoming increasingly recognized as important risk factor for human health in low-, middle-, and high-income countries alike [30]. 

Indoor air pollution is a heterogeneous combination of particles and gases with concentrations affected by infiltrating outdoor particles, emissions from indoor sources, and particles formed indoors through reactions and secondary processes [31,32]. In high-income countries such as Australia, gas and electric cooking appliances along with heating and cooling appliances, furniture, dust re-suspension and indoor combustion of solid fuels (e.g., wood and coal) are significant contributors to indoor air pollution [31,33,34]. In contrast, major sources of indoor air pollution in low- and middle-income countries traditionally involve cooking and heating using solid fuels such as crop residues, dung, or wood in poorly ventilated homes [22,25,29,35,36]. 

It is well reported that in high-income countries, approximately two-thirds of indoor time is spent in the home [37,38,39]. Despite this, there have been few published studies that have quantitatively examined the relationship between indoor air quality (IAQ) and associated cardiometabolic health effects in high-income countries [19,40], although several studies have investigated this relationship in low- and middle-income countries [22,25,29,41,42]. 

The primary aim of this research was to investigate associations between sub-clinical cardiometabolic risk markers with indoor air pollutants in healthy middle-aged adults residing in Perth, Western Australia. Our research hypothesis assumed that acute exposure to a number of indoor air pollutants would be independently and adversely associated with a range of sub-clinical cardiometabolic risk factors.

## 2. Materials and Methods 

### 2.1. Study Design

A cross-sectional study conducted in a cohort of healthy middle-aged adults living in the metropolitan area of Perth, Western Australia.

### 2.2. Study Location and Population

Perth is the capital of Western Australia. It is a typical Australian capital city with a population of approximately 2.1 million people densely situated around the capital [43], although ambient air quality is considered to be of a high standard compared with other Australian and international cities [44]. 

**Figure 1:**
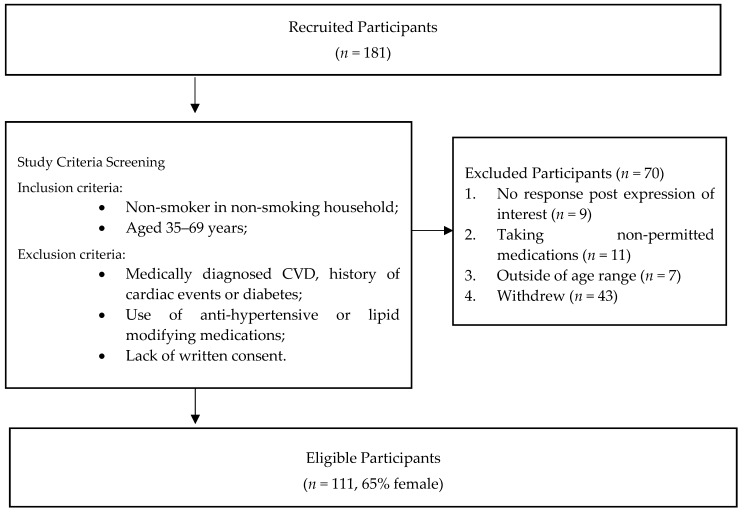
Study population recruitment process.

For this study, we recruited 181 adults, of which 70 were subsequently excluded due to ineligibility with study criteria. The study population selection is depicted at Figure 1. Participants were initially invited to join the study through local radio advertising and a group email to all staff of the hosting University. Further participants were recruited through word-of-mouth. Prior to enrolment, participants were screened for eligibility by a seven-question telephone interview or electronic questionnaire. 

Adults meeting the following criteria were included in the study:

Inclusion criteria:

- Non-smokers living in a non-smoking household.
- Aged between 35 and 69 years.

Exclusion criteria:

- A history of cardiovascular events or medical diagnosis of cardiovascular disease (CVD).
- Medically diagnosed diabetes.
- Use of anti-hypertensive or lipid modifying medications.
- Lack of written consent.

### 2.3. Data Collection

For each participant, data were collected once, although collection involved two stages. Stages 1 and 2 were arbitrarily assigned a number order; however, participants were offered the opportunity to select the stage that was undertaken first. All data collection was carried out by the same investigator following standard protocols. Due to the logistical challenges required to coordinate equipment, investigator availability, and participant preferences, a 14 day limit was set as the maximum time period to complete both stages. Stages 1 and 2 are described in further detail below.

- Stage 1: In-home equipment installation—the measurement of domestic indoor air quality in participants home and ambulatory blood pressure (ABP)/hemodynamic indices over a concurrent 24 h period. Participants were also requested to complete questionnaires related to health and domestic environment and to complete a 24 h time/activity diary during the 24 h IAQ/BP monitoring period.
- Stage 2: Clinic-based health assessment—data relating to baseline health characteristics, cardiometabolic biomarkers and measures, and pulse wave analysis were collected.

### 2.4. In-Home Assessment

Residential indoor and outdoor pollutant concentrations and ambulatory BP and hemodynamic indices were measured over one concurrent 24 h period. All participants were requested to undertake their daily tasks as usual during the monitoring period.

Indoor air pollutants were measured in the main living area and included: Total particulate matter (TPM), PM_10_, PM_4_, PM_2.5_, PM_1_, UFP, formaldehyde (HCHO), total volatile organic compounds (TVOCs), nitrogen dioxide (NO_2_), carbon monoxide (CO) and carbon dioxide (CO_2_). Ancillary measurements included temperature and relative humidity (RH). Due to limitations with equipment availability, outdoor measurements were conducted only for PM (TPM, PM_10_, PM_4_, PM_2.5_ and PM_1_). The in-home assessment was undertaken at a time convenient to participants prior to 4 pm. Due to operational limitations of the instrumentation, UFP measurements were undertaken for 6 hs only, between 4 pm and 10 pm.

Outdoor air monitoring equipment was placed in the closest powered location to the house, under shade for protection from rain and sunlight [32], and away from any combustion sources such as barbeques and driveways [45]. Indoor and outdoor residential sampling was conducted at a height of 1.5 m, which is the approximate breathing zone of a standing adult [45]. Air sampling was undertaken on weekdays and weekends, as recent research has established that mass concentrations of fine (PM_2.5_) and coarse particles (PM_10-2.5_) do not differ in relation to days of the week [46]. 

#### 2.4.1. Measurement of Air Quality and Instrumentation

Air quality was determined using instruments that measure particle mass concentrations (μg/m^3^; DustTrak DRX 8533. TSI Inc., Shoreview, MN, USA), UFP number concentration (particles/cm^3^; P-Trak model 8525. TSI Inc., Shoreview, MN, USA), total volatile organic compounds (TVOC; ppb), carbon dioxide (CO_2_; ppm), carbon monoxide (CO; ppm), nitrogen dioxide (NO_2_; ppm), temperature (°C) and relative humidity (%RH) (Advanced Sense Pro. Gray Wolf Sensing Solutions, Shelton, CT, USA), and formaldehyde (HCHO; μg/m^3^) (Formaldehyde Multimode Monitor FM-801. Gray Wolf Sensing Solutions, Shelton, CT, USA). 

The DustTrak DRX 8533 is a light-scattering laser photometer that simultaneously measures real-time aerosol mass readings for five particle size fractions (TPM, PM_10_, PM_4_, PM_2.5_, PM_1_) [47]. Two DustTrak 8533 instruments were used at each dwelling to simultaneously measure indoor and outdoor air quality for the 24 h monitoring period. Both instruments were pre-set with residential location details before commencing monitoring. The measuring range of the instrument was 1–150 × 10^3^ μg/m^3^ with an accuracy of ±0.1%. Flow rate was factory set at 3.0 L/min. DustTrak was programmed to log data at 5 min intervals for the full 24 hs.

Zero calibration for both instruments was conducted on-site prior to the commencement of data logging to minimize the effect of zero drift. Instruments were factory calibrated for flow rate prior to the commencement of the project, and flow rates were intermittently checked during the course of the project by a calibrated rotameter (TSI LPM-air).

A portable P-Trak 8525 was used to detect and count UFP < 1 μm in real-time. P-Trak displayed the measured particle concentration in units of particles per cubic centimeter (particles/cm^3^), and the instrument’s measuring range was from 0 to 500,000 particles/cm^3^. The flow rate for the sampling was approximately 100 cm^3^/min [48]. The alcohol wick used by P-Trak had a limit of operation of 8 hs at 21 °C before requiring re-saturation [49], and, as such, the instrument was programmed to only log data for a 6 h period. Data were logged at 5 min intervals.

Data from both the DustTrak 8533 and P-Trak 8525 were downloaded using TrakPro data analysis software.

Gaseous pollutants were measured using the Gray Wolf AdvancedSense Pro fitted with a sensor probe measuring TVOCs, CO_2_, CO, NO_2_, RH, and temperature. A separate, supplementary monitor was attached to the AdvancedSense Pro measuring HCHO (Multimode monitor FM-801).

Both instruments were pre-set with residential location details before commencing monitoring and programmed to log data at 30 min intervals.

#### 2.4.2. Measurement of Ambulatory BP and Hemodynamic Indices

An ambulatory BP monitor (ABPM) (Oscar 2, Sun Tech Medical, Inc., Morrisville, NC, USA) was fitted to the left arm of the participant, having been pre-programmed to obtain readings at 30 min intervals for the full 24 hs. The device was programmed to automatically attempt an additional measurement 4 min later if the previous was unsuccessful due to participant movement or positioning. Measurements collected from ABPM included: 24 h central and peripheral systolic BP (SBP); 24 h central and peripheral diastolic BP (DBP); day-time central and peripheral SBP; night-time central and peripheral DBP (all BP measurements were in mmHg); 24 h, day-time and night-time heart rate (beats per minute; bpm); central and peripheral systolic and diastolic nocturnal dip (%), central augmentation index (AIx; %) along with AIx adjusted for heart rate (AI_75_; %); and central and peripheral augmented pressure, pulse pressure and mean arterial pressure (all measured in mmHg). 

Awake and asleep periods were determined from time-activity diaries maintained by participants for the 24 h monitoring period, and used the same method as several other studies [50,51,52].

Following standard protocol described in Parati et al. [52] and O’Brien et al. [51], measurements were deemed valid and included in the final analyses if 70% of the 24 h measurements were obtained, and 20 valid awake and 7 valid asleep measurements were achieved. When < 70% of 24 h readings were achieved, further investigation was undertaken of edited day-time and night-time readings. Day-time or night-time measurements that did not pass validity criteria for the time period (day-time: 20 valid readings; night-time: 7 valid readings) were discarded and excluded from the final analyses by a process described in similar studies [51,53]. All valid day-time and night-time readings were averaged to provide a single day-time and night-time ABP value per study participant [53].

At the end of the 24 h ± 2 hs sampling period, equipment and completed questionnaires were collected from participants’ homes. 

#### 2.4.3. Questionnaires and Time-Activity Diary

Each participant was provided with two questionnaires and a time-activity diary to be completed during the 24 h monitoring.

Participant demographics including address, age, gender and ethnicity (country of birth, parents country of birth), along with information on health and lifestyle behaviors, were gathered by an adapted version of the American Thoracic Society standardized IAQ and health questionnaire that has also been used in several other Australian studies [40,54,55,56]. In the health questionnaire, participants self-reported on factors including smoking status (yes, no), alcohol intake (more or less than two alcoholic drinks per day), medications taken, and comorbidities including conditions such as asthma, chronic obstructive pulmonary disease, kidney disease and thyroid conditions. From the demographic data collected, socioeconomic status was assigned using census-track data collected by the Australian Bureau of Statistics [57] and by using post codes to rank participants homes according to relative socio-economic advantage (low, medium or high).

Participants also completed a questionnaire to report on the characteristics of their domestic environment; a questionnaire which has been used in several previous studies examining IAQ [54,55,56]. Survey questions asked about indoor air pollution sources such as cooking technology used (gas, electric or both), heating (flued and unflued gas, wood, coal, oil, kerosene, electric, or reverse cycle air conditioning) and cooling devices (fans, refrigerated air conditioning, or evaporative air conditioning), floor and wall coverings, renovations (painting, building, renovations in previous three months; yes, no), cleaning habits (frequency and types of products), and garage location (attached to the home by inner door or not attached). 

Additionally, participants reported their time-activity for the 24 h monitoring period, indicating for each two hour period their presence indoors/outdoors, and their presence at home/work/other. Other recorded activities included their sleep schedule (awake time and asleep time) or any situations they consider important (e.g., stressful situations or unusual sleep times). Data collected using this method is consistent with other similar studies [45,58].

### 2.5. Clinic Assessment

Participants attended the Curtin University Clinical Health Suites for a health assessment after having fasted for 12 hs (other than water and regular medications) prior [59].

Participants’ height (m) and weight (kg) were measured in bare feet and wearing light clothing. Weight was measured by a mechanical scale (SECA 762, SECA, Hamburg, Germany) and height was measured by a stadiometer (S + M. Surgical and Medical Products, Preston, VIC, Australia). Body mass index (BMI) was calculated by dividing body weight (kg) by the squared height (m^2^). Waist (cm) and hip circumference (cm) were measured using a non-stretch, retractable tape. After following standard protocol for identifying the level of the natural waist and hips and assuring the tape was level, the hip circumference was recorded. Waist circumference was measured at the end of a participant’s normal expiration. The waist-to-hip ratio was calculated by dividing the waist measurement by the hip measurement [59].

Brachial (peripheral) blood pressure (mmHg) was measured after a 5 min rest in the sitting position. BP recordings on both arms were made one minute apart using an automated monitor (HEM-907, Omron Healthcare, Kyoto, Japan) [60]. The mean of three measures was considered the brachial BP and was calculated independently for both arms.

Two finger stick blood samples were collected using an aseptic technique. These samples were immediately analyzed for lipids (total cholesterol, high density lipoprotein [HDL], low density lipoprotein [LDL], non-HDL, triglycerides, fasting glucose [all in mmol/L], and glycosated haemoglobin [HbA1c; %]). A spot mid-stream urine sample was requested for the determination of albumin (mg/L), creatinine (mmol/L) and albumin/creatinine ratio (ACR, mg/mmol). Lipid levels, HbA1c and urine analysis was conducted using the appropriate reagent containing cassettes of the fully automated biochemistry analysis system (Alere Afinion AS100, Waltham, MASS, USA).

Fasting glucose was analyzed by a hand-held Accu-Chek Blood Glucose Meter (Roche Diabetes Care, Mannheim, Germany) using blood drawn from a finger stick test. 

#### Pulse Wave Analysis and Pulse Wave Velocity

Central BP and wave-forms were non-invasively measured with the participant in a supine position (SphygmoCor EM3 XCEL, AtCor Medical Pty, West Ryde, Australia). Carotid–femoral pulse wave velocity (PWV) was determined by examining the arterial waveforms of the carotid and femoral arteries, as well as the time delay measured between the feet of the two waveforms. The distance covered by the waves was established as 80% of the distance between the two recording sites. PWV was calculated by SphygmoCor proprietary software as distance/time delay in m/s [61].

### 2.6. Data Management

Each participant was given a unique alpha-numeric identification number to secure personal data. Data will be managed and stored according to the Australian Code for the Responsible Conduct of Research. Electronic files will be backed up, maintained, and stored on Curtin University’s secure Research drive, and hard copy original material including questionnaires and time-activity data will be stored in numerical order, in a locked compactus, in a secure entry room. Access to the study data will be restricted, and participant files will be maintained in storage for a period of seven years after completion of the study.

### 2.7. Statistical Analysis

#### 2.7.1. Sample Size and Effect

Assuming a conventional Cohen’s medium effect size of 15%, a sample size of 110 was calculated by G power to achieve 80% power for testing both the overall significance of the multivariable linear regression model and an individual effect attributed to the residential IAQ on cardiometabolic risk markers at a significance level of 5%. This medium effect size is equivalent to testing an overall multiple correlation of 0.13 and a partial correlation of 0.12, which are comparable with preliminary results from a recent Australian study (*n* = 63) [40]. The final sample size obtained was further verified by Green’s formula for general practical applications using a multivariable regression analysis [62].

#### 2.7.2. Data Analysis

Descriptive statistics were calculated to describe the profile and characteristics of each study participant and included demographic characteristics, some health characteristics, air pollutant characteristics and home environment characteristics. Further descriptive statistics were computed for all exposure and health outcomes. Values are expressed as mean (± standard deviation) for continuous variables and number (percentage) for categorical variables. The normality of all continuous data was assessed using histograms, boxplots, a normal Q–Q plot, and skewness and kurtosis coefficients. 

Paired samples t-tests were undertaken to evaluate differences between clinic and ambulatory blood pressure measurements. Simple associations between every outcome variable and all 24 h average indoor pollutant concentrations were examined by using the Pearson *r* coefficient.

Linear mixed-effect models were then applied to investigate the associations between sub-clinical end points of interest and indoor air pollutants. Saturated models were adjusted for potential covariates selected a priori and on the basis of similar studies previously reported in the literature [63,64,65]. Single pollutant exposure models were controlled for age (continuous), gender (nominal; male, female), BMI (continuous), waist–hip ratio (continuous) and socio-economic status (ordinal; low, medium, high). Univariable tests were carried out to explore associations between explanatory variables and each outcome (dependent) variable, and to identify which of the explanatory variables were individually associated with the dependent variables. We assessed residual confounding by waist circumference, ethnicity and alcohol consumption by adding variables individually to the base model to evaluate their influence on the results [17]. Effect estimates did not significantly alter with their inclusion, so they were not included in the final model.

One participant was considered to have a contentious health and lifestyle profile (borderline hypertension; >2 alcoholic drinks per day). However, other cardiometabolic biomarkers were in the expected range. A sensitivity analysis was undertaken with this participant excluded; however, no significant impact to the final results was observed following their exclusion. 

The final results were reported by mean change in the outcome variable corresponding to a one interquartile range (IQR) increase of exposure to each indoor air pollutant concentration, along with its 95% confidence interval.

A *p*-value ≤ 0.05 was considered statistically significant. Analyses were performed using IBM SPSS software (Version 24.0. IBM Corp, Armonk, NY, USA.)

### 2.8. Ethics and Consents

This study protocol was approved by Curtin University, Human Research Ethics Committee (HRE2016-0308). Upon enrolment, participants were provided with a study information sheet which outlined the study procedure and the voluntary nature of their involvement. Recruits were provided the opportunity to ask research team further questions, following which full written, informed consent was obtained. Details of the research team are contained on the participant information sheet. Findings from this study will be disseminated locally and at international meetings and will be published in open access peer reviewed journals. 

## 3. Discussion

This cross-sectional study of middle-aged, healthy individuals was designed to characterize the associations between exposure to residential indoor air pollution and selected intermediate cardiometabolic outcomes in a high-income setting. By using objective measurement tools for the ascertainment of both exposure and health-related endpoints, we hoped to minimize possible measurement errors that are likely associated with the use of estimated or inferred data. 

While previous studies have focused on associations between ambient air pollution and human health, there is increasing evidence to suggest a role for indoor air pollution [66], and indeed it has been estimated that up to 30% of the burden of disease from exposure to air pollution can be attributed to indoor-generated particles [67]. Indoor air pollution arises from numerous different sources along with post-emission formation and processes, and as such, indoor air pollutants can retain complex compositions and toxicities, with some similarities but also differences to outdoor air pollution [33].

A limitation of previous studies that have reported on relationships between indoor and outdoor air pollution and health [68] is that the exposure of the individual was not directly observed; rather, estimations were made by extrapolating an exposure value or exposure variation from a central fixed monitoring site to the entire population of the study area [19,69,70,71,72,73,74]. However, this approach may have resulted in exposure misclassification [68,75] and potentially biased estimates, as characteristics of individual exposure may be wrongly inferred from estimated characteristics of the collective population [68]. 

Similar limitations exist when considering individual level clinical cardiometabolic effects with outcomes such as ischemic heart disease, stroke, and type 2 diabetes mellitus commonly being derived through record linkage procedures, hospital admission/discharge registries, or by self-reporting [72,73,76,77,78,79]. Such data are then ordinarily linked to the ecologically-derived exposure data to ascertain the directionality of a relationship, leading to opportunities for the possibility of misclassification based on incorrect assumptions about individual-level pollutant characteristics [68]. Acknowledging these limitations, significant associations have been described between ambient air quality and increased cardiometabolic risk at air pollution concentrations below recommended international standards [12,80,81].

Similar to other studies [29,35], this current study had several limitations. 

Given the cross-sectional design, we were not able to establish temporality between exposure and health outcomes. Though cardiometabolic measures were concurrently determined (within 14 days) to air pollution exposure, based on our associations, it was not possible to infer causation. Additionally, although there was up to a 14 day gap between exposure and health outcome measurements, we considered our exposure assessment to be a plausible representation of individual habitual exposure to air pollutants in the residential environment under normally occupied conditions. Additionally, without significant variation in individual behavior and residential circumstances, it is unlikely that the 14 day lag would have had appreciable influence on the results. 

Though we adjusted for important and well-established confounders, residual confounding was still a possibility due to unmeasured or incompletely measured factors. These factors include potential effect modifiers such as genetic pre-disposition and exercise or dietary factors that can affect some of the measured cardiometabolic markers. These are limitations that have been reported in other similar studies [29,35].

However, there were also several notable strengths of our study. All exposure and health metrics were directly measured without relying on surrogates or self-report, and several of these markers are considered robust measures related to cardiometabolic risk. In our study, we examined several measures of arterial stiffness, including pulse wave velocity (PWV) which is considered a reliable predictor of cardiovascular events and outcomes [82]. Additionally, HbA1c is a measure that captures chronic glucose exposure and is not influenced by short-term dietary intake. It is considered a reliable measure of diabetic risk [83].

Finally, the findings of this research will contribute to a more thorough understanding of the relationship between measured domestic IAQ and selected functional sub-clinical endpoints related to cardiometabolic risk in high-income countries. Potential outcomes of this research may include providing direction for future research to further understand the intermediate mechanistic pathways by which air pollution might contribute to cardiometabolic risk.

The effective dissemination of findings from this research will raise awareness and potentially provide some insight regarding the relationship between exposure and cardiometabolic response, particularly at low and very low concentrations of airborne air pollutants, and contribute to current understandings of whether thresholds exist for ‘safe’ exposure to indoor airborne air pollutants.

## 4. Conclusions

This study provides a standardized approach to the study of sub-clinical cardiometabolic health effects that are related to exposure to indoor air pollution in a high-income setting. Given the ubiquitous nature of indoor air pollution, objective and reliable methods to measure exposure will assist in the quantification of its effects on health. Whilst further longitudinal work of a prospective design is required to confirm the causal association between indoor air pollution and adverse sub-clinical cardiometabolic outcomes, the findings from this study may provide important insights into the mechanistic pathways by which exposure to indoor air pollution affects health outcomes and, thus, provide the basis for future research.

## References

[1] Cosselman K.E., Navas-Acien A., Kaufman J.D. (2015). Environmental factors in cardiovascular disease. Nat. Revs. Cardiol..

[2] Munzel T., Sorensen M., Gori T., Schmidt F.P., Rao X., Brook F.R., Chen L.C., Brook R.D., Rajagopalan S. (2017). Environmental stressors and cardio-metabolic disease: Part ii-mechanistic insights. Eur. Heart J..

[3] Munzel T., Sorensen M., Gori T., Schmidt F.P., Rao X., Brook J., Chen L.C., Brook R.D., Rajagopalan S. (2017). Environmental stressors and cardio-metabolic disease: Part i-epidemiologic evidence supporting a role for noise and air pollution and effects of mitigation strategies. Eur. Heart J..

[4] Brook R.D., Bard R.L., Burnett R.T., Shin H.H., Vette A., Croghan C., Phillips M., Rodes C., Thornburg J., Williams R. (2011). Differences in blood pressure and vascular responses associated with ambient fine particulate matter exposures measured at the personal versus community level. Occup. Environ. Med..

[5] Dvonch T.J., Kannan J.S., Schulz J.A., Keeler L.G., Mentz D.G., House D.J., Benjamin D.A., Max D.P., Bard D.R., Brook D.R. (2009). Acute effects of ambient particulate matter on blood pressure: Differential effects across urban communities. Hypertension.

[6] Lenters V., Uiterwaal C.S., Beelen R., Bots M.L., Fischer P., Brunekreef B., Hoek G. (2010). Long-term exposure to air pollution and vascular damage in young adults. Epidemiology.

[7] Mehta A.J., Zanobetti A., Koutrakis P., Mittleman M.A., Sparrow D., Vokonas P., Schwartz J. (2014). Associations between short-term changes in air pollution and correlates of arterial stiffness: The veterans affairs normative aging study, 2007–2011. Am. J. Epidemiol..

[8] Shanley P.R., Hayes B.R., Cromar R.K., Ito R.K., Gordon R.T., Ahn R.J. (2016). Particulate air pollution and clinical cardiovascular disease risk factors. Epidemiology.

[9] Yang B.-Y., Bloom M.S., Markevych I., Qian Z., Vaughn M.G., Cummings-Vaughn L.A., Li S., Chen G., Bowatte G., Perret J.L. (2018). Exposure to ambient air pollution and blood lipids in adults: The 33 communities chinese health study. Environ. Int..

[10] Yang B.-Y., Qian Z., Howard S.W., Vaughn M.G., Fan S.-J., Liu K.-K., Dong G.-H. (2018). Global association between ambient air pollution and blood pressure: A systematic review and meta-analysis. Environ. Pollut..

[11] Brook R.D., Newby D.E., Rajagopalan S. (2017). Air pollution and cardiometabolic disease: An update and call for clinical trials. Am. J. Hypertens..

[12] Bourdrel T., Bind M.A., Bejot Y., Morel O., Argacha J.F. (2017). Cardiovascular effects of air pollution. Arch. Cardiovasc. Dis..

[13] Kelly F.J., Fussell J.C. (2015). Air pollution and public health: Emerging hazards and improved understanding of risk. Environ. Geochem. Health.

[14] Uzoigwe J.C., Prum T., Bresnahan E., Garelnabi M. (2013). The emerging role of outdoor and indoor air pollution in cardiovascular disease. N. Am. J. Med. Sci..

[15] Brook R.D., Weder A.B., Rajagopalan S. (2011). “Environmental hypertensionology” the effects of environmental factors on blood pressure in clinical practice and research. J. Clin. Hypertens..

[16] Giorgini P., Di Giosia P., Grassi D., Rubenfire M., Brook R.D., Ferri C. (2016). Air pollution exposure and blood pressure: An updated review of the literature. Curr. Pharm. Des..

[17] Auchincloss A., Roux A., Dvonch J., Brown P., Barr R., Daviglus M., Goff D., Kaufman J., O’Neill M. (2008). Associations between recent exposure to ambient fine particulate matter and blood pressure in the multi-ethnic study of atherosclerosis (mesa). Environ. Health Perspect..

[18] Ibald-Mulli A., Stieber J., Wichmann H.E., Koenig W., Peters A. (2001). Effects of air pollution on blood pressure: A population-based approach. Am. J. Public Health.

[19] Honda T., Pun V.C., Manjourides J., Suh H. (2018). Associations of long-term fine particulate matter exposure with prevalent hypertension and increased blood pressure in older americans. Environ. Res..

[20] Harrabi I., Rondeau V., Dartigues J.-F., Tessier J.-F., Filleul L. (2006). Effects of particulate air pollution on systolic blood pressure: A population-based approach. Environ. Res..

[21] Madsen C., Nafstad P. (2006). Associations between environmental exposure and blood pressure among participants in the oslo health study (hubro). Eur. J. Epidemiol..

[22] Baumgartner J., Carter E., Schauer J.J., Ezzati M., Daskalopoulou S.S., Valois M.F., Shan M., Yang X. (2018). Household air pollution and measures of blood pressure, arterial stiffness and central haemodynamics. Heart.

[23] Chuang K.-J., Yan Y.-H., Cheng T.-J. (2010). Effect of air pollution on blood pressure, blood lipids, and blood sugar: A population-based approach. J. Occup. Environ. Med..

[24] Chuang K.-J., Yan Y.-H., Chiu S.-Y., Cheng T.-J. (2011). Long-term air pollution exposure and risk factors for cardiovascular diseases among the elderly in taiwan. Occup. Environ. Med..

[25] Rajkumar S., Young B.N., Clark M.L., Benka-Coker M.L., Bachand A.M., Brook R.D., Nelson T.L., Volckens J., Reynolds S.J., L’orange C. (2019). Household air pollution from biomass-burning cookstoves and metabolic syndrome, blood lipid concentrations, and waist circumference in honduran women: A cross-sectional study. Environ. Res..

[26] Yitshak Sade F.M., Kloog F.I., Liberty F.I., Schwartz F.J., Novack F.V. (2016). The association between air pollution exposure and glucose and lipids levels. J. Clin. Endocrinol. Metab..

[27] Xiao S., Liu R., Wei Y., Feng L., Lv X., Tang F. (2016). Air pollution and blood lipid markers levels: Estimating short and long-term effects on elderly hypertension inpatients complicated with or without type 2 diabetes. Environ. Pollut..

[28] Liu C., Yang C., Zhao Y., Ma Z., Bi J., Liu Y., Meng X., Wang Y., Cai J., Kan H. (2016). Associations between long-term exposure to ambient particulate air pollution and type 2 diabetes prevalence, blood glucose and glycosylated hemoglobin levels in china. Environ. Int..

[29] Rajkumar S., Clark M.L., Young B.N., Benka-Coker M.L., Bachand A.M., Brook R.D., Nelson T.L., Volckens J., Reynolds S.J., L’Orange C. (2018). Exposure to household air pollution from biomass-burning cookstoves and hba1c and diabetic status among honduran women. Indoor Air.

[30] World Health Organisation (2010). Who: Guidelines for Indoor Air Quality: Selected Pollutants.

[31] Morawska L., Afshari A., Bae G.N., Buonanno G., Chao C.Y.H., Hänninen O., Hofmann W., Isaxon C., Jayaratne E.R., Pasanen P. (2013). Indoor aerosols: From personal exposure to risk assessment. Indoor Air.

[32] Bhangar S., Mullen N., Hering S., Kreisberg N., Nazaroff W. (2011). Ultrafine particle concentrations and exposures in seven residences in northern california. Indoor Air.

[33] Morawska L., Ayoko G.A., Bae G.N., Buonanno G., Chao C.Y.H., Clifford S., Fu S.C., Hanninen O., He C., Isaxon C. (2017). Airborne particles in indoor environment of homes, schools, offices and aged care facilities: The main routes of exposure. Environ. Int..

[34] Wallace L. (2006). Indoor sources of ultrafine and accumulation mode particles: Size distributions, size-resolved concentrations, and source strengths. Aerosol Sci. Technol..

[35] Clark S.N., Schmidt A.M., Carter E.M., Schauer J.J., Yang X., Ezzati M., Daskalopoulou S.S., Baumgartner J. (2019). Longitudinal evaluation of a household energy package on blood pressure, central hemodynamics, and arterial stiffness in China. Environ. Res..

[36] Young B.N., Clark M.L., Rajkumar S., Benka-Coker M.L., Bachand A., Brook R.D., Nelson T.L., Volckens J., Reynolds S.J., L’Orange C. (2019). Exposure to household air pollution from biomass cookstoves and blood pressure among women in rural honduras: A cross-sectional study. Indoor Air.

[37] Brasche S., Bischof W. (2005). Daily time spent indoors in german homes–baseline data for the assessment of indoor exposure of german occupants. Int. J. Hyg. Environ. Health..

[38] Lai H.K., Kendall M., Ferrier H., Lindup I., Alm S., Hänninen O., Jantunen M., Mathys P., Colvile R., Ashmore M.R. (2004). Personal exposures and microenvironment concentrations of PM_2.5_, VOC, NO_2_ and CO in Oxford, UK. Atmos. Environ..

[39] Leech J.A., Nelson W.C., Burnett R.T., Aaron S., Raizenne M.E. (2002). It’s about time: A comparison of canadian and american time-activity patterns. J. Expo. Anal. Environ. Epidemiol..

[40] Rumchev K., Soares M., Zhao Y., Reid C., Huxley R. (2018). The association between indoor air quality and adult blood pressure levels in a high-income setting. Int. J. Environ. Res. Public Health.

[41] Balmes J.R. (2019). Household air pollution from domestic combustion of solid fuels and health. J. Allergy Clin. Immunol..

[42] Reisen F., Meyer C.P., Keywood M.D. (2013). Impact of biomass burning sources on seasonal aerosol air quality. Atmos. Environ..

[43] Australian Bureau of Statistics Greater Perth; Western Australia. https://itt.abs.gov.au/itt/r.jsp?RegionSummary&region=5GPER&dataset=ABS_REGIONAL_ASGS&geoconcept=REGION&measure=MEASURE&datasetASGS=ABS_REGIONAL_ASGS&datasetLGA=ABS_REGIONAL_LGA&regionLGA=REGION&regionASGS=REGION.

[44] Government of Western Australia, Environmental Protection Authority Factor Guidelines and Technical Guidance: Air. http://epa.wa.gov.au/node/193.

[45] Wheeler A.J., Xu X., Kulka R., You H., Wallace L., Mallach G., Ryswyk K.V., MacNeill M., Kearney J., Rasmussen P.E. (2011). Windsor, ontario exposure assessment study: Design and methods validation of personal, indoor, and outdoor air pollution monitoring. J. Air Waste Manag. Assoc..

[46] Yoda Y., Tamura K., Shima M. (2017). Airborne endotoxin concentrations in indoor and outdoor particulate matter and their predictors in an urban city. Indoor Air.

[47] TSI Dust Trak Aerosol Monitor-Model 8533, Operation and Service Manual. http://www.tsi.com/uploadedFiles/_Site_Root/Products/Literature/Manuals/8533-8534-DustTrak_DRX-6001898-web.pdf.

[48] Akbar-khanzadeh F., Ames A., Bisesi M., Milz S., Czajkowski K., Kumar A. (2012). Particulate matter (pm) exposure assessment—Horizontal and vertical pm profiles in relation to agricultural activities and environmental factors in farm fields. J. Occup. Environ. Hyg..

[49] TSI P-Trak Ultrafine Particle Counter 8525-Operation and Service Manual. http://www.tsi.com/uploadedFiles/_Site_Root/Products/Literature/Manuals/Model-8525-P-Trak-1980380.pdf.

[50] Sun Z.C., Mukherjee B., Brook R.D., Gatts G., Yang F., Sun Q., Brook J.R., Fan Z., Rajagopalan S. (2013). Air-pollution and cardiometabolic diseases (aircmd): A prospective study investigating the impact of air pollution exposure and propensity for type ii diabetes. Sci. Total Environ..

[51] O’Brien E., Parati G., Stergiou G., Asmar R., Beilin L., Bilo G., Clement D., de la Sierra A., de Leeuw P., Dolan E. (2013). European society of hypertension position paper on ambulatory blood pressure monitoring. J. Hypertens..

[52] Parati G., Stergiou G., O’Brien E., Asmar R., Beilin L., Bilo G., Clement D., de la Sierra A., de Leeuw P., Dolan E. (2014). European society of hypertension practice guidelines for ambulatory blood pressure monitoring. J. Hypertens..

[53] Andreadis E.A., Agaliotis G., Kollias A., Kolyvas G., Achimastos A., Stergiou G.S. (2016). Night-time home versus ambulatory blood pressure in determining target organ damage. J. Hypertens..

[54] Rumchev K., Spickett J., Bulsara M., Phillips M., Stick S. (2004). Association of domestic exposure to volatile organic compounds with asthma in young children. Thorax.

[55] Rumchev K.B., Spickett J.T., Bulsara M.K., Phillips M.R., Stick S.M. (2002). Domestic exposure to formaldehyde significantly increases the risk of asthma in young children. Eur. Respir. J..

[56] Zhang G., Spickett J., Rumchev K., Lee A.H., Stick S. (2004). Snoring in primary school children and domestic environment: A perth school based study. Respir. Res..

[57] Australian Government Diabetes. http://www.health.gov.au/internet/main/publishing.nsf/Content/chronic-diabetes#ris.

[58] Fuller C.H., Patton A.P., Lane K., Laws M.B., Marden A., Carrasco E., Spengler J., Mwamburi M., Zamore W., Durant J.L. (2013). A community participatory study of cardiovascular health and exposure to near-highway air pollution: Study design and methods. Rev. Environ. Health.

[59] Anderson G.L., Manson J., Wallace R., Lund B., Hall D., Davis S., Shumaker S., Wang C.-Y., Stein E., Prentice R.L. (2003). Implementation of the women’s health initiative study design. Ann. Epidemiol..

[60] Ji H., Xiong J., Yu S., Chi C., Fan X., Bai B., Zhou Y., Teliewubai J., Lu Y., Xu H. (2017). Northern shanghai study: Cardiovascular risk and its associated factors in the chinese elderly—A study protocol of a prospective study design. BMJ Open.

[61] de Vos L.C., Boersema J., Hillebrands J.L., Schalkwijk C.G., Meerwaldt R., Breek J.C., Smit A.J., Zeebregts C.J., Lefrandt J.D. (2017). Diverging effects of diabetes mellitus in patients with peripheral artery disease and abdominal aortic aneurysm and the role of advanced glycation end-products: Artery study—protocol for a multicentre cross-sectional study. BMJ Open.

[62] Green S.B. (1991). How many subjects does it take to do a regression analysis. Multivar. Behav. Res..

[63] Chan S., Bergen S., Szpiro A., Deroo L., London S., Marshall J., Kaufman J., Sandler D. (2015). Long-term air pollution exposure and blood pressure in the sister study. Environ. Health Perspect. (Online).

[64] Adar S.D., Chen Y.H., D’Souza J.C., O’Neill M.S., Szpiro A.A., Auchincloss A.H., Park S.K., Daviglus M.L., Diez Roux A.V., Kaufman J.D. (2018). Longitudinal analysis of long-term air pollution levels and blood pressure: A cautionary tale from the multi-ethnic study of atherosclerosis. Environ. Health Perspect..

[65] Sanchez-Inigo L., Navarro-Gonzalez D., Pastrana-Delgado J., Fernandez-Montero A., Martinez J.A. (2016). Association of triglycerides and new lipid markers with the incidence of hypertension in a spanish cohort. J. Hypertens..

[66] Morawska L. (2010). Indoor air quality and health. Air Qual. Clim. Chang..

[67] Morawska L., He C., Hitchins J., Mengersen K., Gilbert D. (2003). Characteristics of particle number and mass concentrations in residential houses in brisbane, australia. Atmos. Environ..

[68] Wilson J.G., Kingham S., Pearce J., Sturman A.P. (2005). A review of intraurban variations in particulate air pollution: Implications for epidemiological research. Atmos. Environ..

[69] Chang L.-T., Chuang K.-J., Yang W.-T., Wang V.-S., Chuang H.-C., Bao B.-Y., Liu C.-S., Chang T.-Y. (2015). Short-term exposure to noise, fine particulate matter and nitrogen oxides on ambulatory blood pressure: A repeated-measure study. Environ. Res..

[70] Dabass A., Talbott E.O., Venkat A., Rager J., Marsh G.M., Sharma R.K., Holguin F. (2016). Association of exposure to particulate matter (pm2.5) air pollution and biomarkers of cardiovascular disease risk in adult nhanes participants (2001–2008). Int. J. Hyg. Environ. Health..

[71] Foraster M., Kunzli N., Aguilera I., Rivera M., Agis D., Vila J., Bouso L., Deltell A., Marrugat J., Ramos R. (2014). High blood pressure and long-term exposure to indoor noise and air pollution from road traffic. Environ. Health Perspect..

[72] Zanobetti A., Schwartz J. (2005). The effect of particulate air pollution on emergency admissions for myocardial infarction: A multicity case-crossover analysis. Environ. Health Perspect..

[73] Pope C.A., Turner M.C., Burnett R.T., Jerrett M., Gapstur S.M., Diver W.R., Krewski D., Brook R.D. (2015). Relationships between fine particulate air pollution, cardiometabolic disorders, and cardiovascular mortality. Circ. Res..

[74] Liu S., Brook R.D., Huang W., Fan Z., Xu H., Wu R., Sun Z., Zhao X., Ruan Y., Yan J. (2017). Extreme levels of ambient air pollution adversely impact cardiac and central aortic hemodynamics: The aircmd-china study. J. Am. Soc. Hypertens..

[75] Hoek G., Krishnan R.M., Beelen R., Peters A., Ostro B., Brunekreef B., Kaufman J.D. (2013). Long-term air pollution exposure and cardio- respiratory mortality: A review. Environ. Health.

[76] Eze I., Schaffner E., Fischer E., Schikowski T., Adam M., Imboden M., Tsai M., Carballo D., Von Eckardstein A., Kunzli N. (2014). Long-term air pollution exposure and diabetes in a population-based swiss cohort. Environ. Int..

[77] Milojevic A., Wilkinson P., Armstrong B., Bhaskaran K., Smeeth L., Hajat S. (2014). Short-term effects of air pollution on a range of cardiovascular events in england and wales: Case-crossover analysis of the minap database, hospital admissions and mortality. Heart.

[78] Stafoggia M., Samoli E., Alessandrini E., Cadum E., Ostro B., Berti G., Faustini A., Jacquemin B., Linares C., Pascal M. (2013). Short-term associations between fine and coarse particulate matter and hospitalizations in southern europe: Results from the med-particles project. Environ. Health Perspect..

[79] Brook R.D., Cakmak S., Turner M.C., Brook J.R., Crouse D.L., Peters P.A., van Donkelaar A., Villeneuve P., Brion O., Jerrett M. (2013). Long-term fine particulate matter exposure and mortality from diabetes in canada. Diabetes Care.

[80] Brook R.D., Rajagopalan S., Arden Pope C., Brook J.R., Bhatnagar A., Diez-Roux A.V., Holguin F., Hong Y., Luepker R.V., Mittleman M.A. (2010). Particulate matter air pollution and cardiovascular disease: An update to the scientific statement from the american heart association. (aha scientific statements) (report). Circulation.

[81] Pinault L., Tjepkema M., Crouse D.L., Weichenthal S., van Donkelaar A., Martin R.V., Brauer M., Chen H., Burnett R.T. (2016). Risk estimates of mortality attributed to low concentrations of ambient fine particulate matter in the canadian community health survey cohort. Environ. Health Glob. Access Sci. Source.

[82] Chirinos J., Zambrano J.P., Veerani A., Schob A., Perez G., Mendez A., Chakko S. (2005). Aortic pressure augmentation predicts adverse cardiovascular events in patients with established coronary artery disease. Eur. Heart J..

[83] The Internaltional Expert Committee (2009). International expert committee report on the role of the a1c assay in the diagnosis of diabetes. Diabetes Care.

